# Photoprogrammed Multifunctional Optoelectronic Synaptic Transistor Arrays Based on Photosensitive Polymer‐Sorted Semiconducting Single‐Walled Carbon Nanotubes for Image Recognition

**DOI:** 10.1002/advs.202401794

**Published:** 2024-06-03

**Authors:** Nianzi Sui, Yixi Ji, Min Li, Fanyuan Zheng, Shuangshuang Shao, Jiaqi Li, Zhaoxin Liu, Jinjian Wu, Jianwen Zhao, Lain‐Jong Li

**Affiliations:** ^1^ School of Nano‐Tech and Nano‐Bionics University of Science and Technology of China No. 398 Ruoshui Road, Suzhou Industrial Park Suzhou Jiangsu Province 215123 P. R. China; ^2^ Division of Nanodevices and Related Nanomaterials Suzhou Institute of Nano‐Tech and Nano‐Bionics Chinese Academy of Sciences No. 398 Ruoshui Road, Suzhou Industrial Park Suzhou Jiangsu Province 215123 P. R. China; ^3^ School of Artificial Intelligence Xidian University Xi'an 710071 P. R. China; ^4^ Department of Mechanical Engineering The University of Hong Kong Pokfulam Road Hong Kong 999077 P. R. China

**Keywords:** broadband response, flexible optoelectronic synaptic devices, multilevel storage, photosensitive conjugated polymer, spiking neural network

## Abstract

The development of neuromorphic optoelectronic systems opens up the possibility of the next generation of artificial vision. In this work, the novel broadband (from 365 to 940 nm) and multilevel storage optoelectronic synaptic thin‐film transistor (TFT) arrays are reported using the photosensitive conjugated polymer (poly[(9,9‐dioctylfluorenyl‐2,7‐diyl)‐co‐(bithiophene)], F8T2) sorted semiconducting single‐walled carbon nanotubes (sc‐SWCNTs) as channel materials. The broadband synaptic responses are inherited to absorption from both photosensitive F8T2 and sorted sc‐SWCNTs, and the excellent optoelectronic synaptic behaviors with 200 linearly increasing conductance states and long retention time > 10^3^ s are attributed to the superior charge trapping at the AlO_x_ dielectric layer grown by atomic layer deposition. Furthermore, the synaptic TFTs can achieve *I*_On_/*I*_Off_ ratios up to 10^6^ and optoelectronic synaptic plasticity with the low power consumption (59 aJ per single pulse), which can simulate not only basic biological synaptic functions but also optical write and electrical erase, multilevel storage, and image recognition. Further, a novel Spiking Neural Network algorithm based on hardware characteristics is designed for the recognition task of Caltech 101 dataset and multiple features of the images are successfully extracted with higher accuracy (97.92%) of the recognition task from the multi‐frequency curves of the optoelectronic synaptic devices.

## Graphical abstract

The authors selectively isolated from commercial SWCNTs using the photosensitive conjugated polymers, and the optoelectronic synaptic transistors are successfully constructed with low power consumption (59 aJ per spike) and excellent storage characteristics   for simulation of neuromorphic vision systems (the accuracy ≈97.92%). Their SWCNT optoelectronic synaptic transistors do not require the introduction of any additional photosensitive materials and exhibit excellent optoelectronic properties.

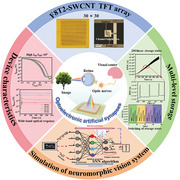

## 1 Introduction

Human brain is a highly efficient neural network that integrates storage and computation, and has a high degree of autonomous learning ability, so the research and development of brain‐like computers has become an urgent need for future AI and robotic technologies. Synapse is an important unit of information transmission and processing in the human brain, and the development of artificial synaptic devices has become a crucial step in the construction of artificial neural networks.^[^ ^1^ ^]^ Faced with the rapid development in artificial vision, the demand for machine vision systems with real‐time image recognition and intelligent analysis is increasing in human life.^[^ ^2^ ^]^ In the human visual system, light receptors in the retinal capture motion and image information and transmit it to the visual cortex of the brain for processing and storage.^[^ ^3^ ^]^ Therefore, optical neuromorphic devices with integrated storage and parallel processing capabilities inspired by human vision are the most critical element. To make the machine or artificial vision system powerful, several important features for optical neuromorphic devices are required including broad‐band and sensitive photo‐detection, low power‐consumption, low latency, and high scalability,^[^ ^4^ ^]^ reliable and reproducible memory states, and long‐term plasticity.

Recently, different optical neuromorphic devices have been reported based on diverse materials, including organic materials,^[^ ^5^ ^]^ perovskites,^[^ ^6^ ^]^ and 2D and 1D materials.^[^ ^7^ ^]^ In brief, many optoelectronic synaptic devices have different issues, such as short recognizable wavelength range, high operating voltage, not reproducible memory (plasticity) states, complicated fabrication processes, and other drawbacks, making it difficult to apply in practice. On the other hand, in biological synapses, the plasticity of the asymmetric behavior associated with signal direction is <15%. Therefore, hardware‐implemented forward inference neural networks require artificial synapses exhibiting a wide range of tunable conductance for high recognition accuracy and analogue computation. Notable progress in optical synaptic devices has been made by Sun et al.^[^ ^8^ ^]^ where a memory device based on 2D ReS_2_ shows wavelength recognition from 405 to 785 nm, which can acquire over 128 distinct storage states. However, most of other optical memristors are difficult to present symmetric conductance changes due to uncontrollable charge relaxation dynamics.

With the improvement of purity, quality as well as scalable fabrication technologies, carbon nanotubes have become a near‐ideal semiconductor material for transistor fabrication^[^ ^9^ ^]^ and large‐scale neuromorphic systems. Zhang et al.^[^ ^10^ ^]^ have adopted carbon nanotubes with PVA/SiO_2_ hybrid plasmonic thin film dielectric layer to realize synaptic functions achieving an ultra‐low power consumption of 360 aJ, a level much lower than that of the human brain. It has also been reported that conjugated polymers can be spontaneously wrapped on semiconducting single walled carbon nanotubes (sc‐SWCNTs) and efficiently sort sc‐SWCNTs, and the polymer encapsulation prevents charge dissipation in SWCNT devices,^[^ ^11^ ^]^ thus offering the possibility of developing high‐performance and low‐cost SWCNT optoelectronic synaptic devices.^[^ ^12^ ^]^

In this work, we report the photo‐programmed multifunctional optoelectronic synaptic transistor arrays with photosensitive polymer (poly[(9,9‐dioctylfluorenyl‐2,7‐diyl)‐co‐(bithiophene)] (F8T2))‐sorted sc‐SWCNTs as the active layer using a scalable preparation method. The SWCNT optoelectronic synaptic transistor exhibits a high *I*_On_/*I*_Off_ ratio (2 × 10^6^) at a low operating voltage (from −1.5 to 1 V). Specifically, the designed optoelectronic devices can realize terrific broadband optical synaptic feature (from 365 to 940 nm), multilevel storages (200 conductance states) and easy synaptic state‐switching, which is attributed to the photon‐induced charge transfer between photosensitive F8T2 to sc‐SWCNTs, and the charge trapping of the AlO_x_ dielectrics grown by ALD at 120 °C. On the basis of our broadband optoelectronic synaptic characteristics, we developed a novel Spiking Neural Network (SNN) algorithm to carry out the recognition task of Caltech 101 dataset and accomplish more quickly (only ≈70 epochs) and accurately (up to 97.92%) the recognition task of the multiple feature images. Our proposed devices exhibit wide‐range recognition, multi‐level storage, storage state switching, and successfully mimics a variety of functions of the human eye, paving a promising route for the construction of artificial visual systems.

## 2 Materials Characterization and Electrical Properties of F8T2‐SWCNT Flexible Carbon Nanotube Transistors

**Figure 1:**
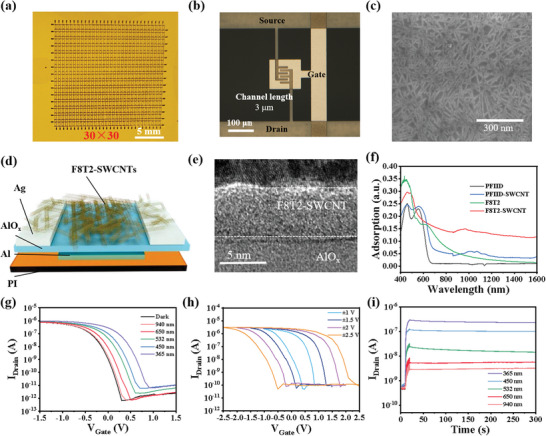
The optical images of a) the 30 × 30 SWCNT TFT array and b) a printed SWCNT TFT. c) SEM image of sc‐SWCNTs deposited in the device channels. d) The structure schematic diagram of the bottom‐gate and bottom‐contact SWCNT TFT device. e) The HRTEM image of cross‐sectional F8T2‐SWCNT on AlO_x_. f) UV–Vis−NIR absorption spectra of the F8T2, F8T2‐SWCNT, PFIID, and PFIID‐SWCNT films. g) Transfer characteristic curves of the broadband optoelectronic synaptic transistor under dark condition, and under light illumination. h) *I*_DS_‐*V*_GS_ memory hysteresis loops under different control gate voltages. i) The *I*_DS_ stimulated by 10 light pulses of different wavelengths with the light power intensity of 6 mW cm^−2^, a width of 0.5 s, and the pulse interval of 0.5 s. *V*_DS_ is set to −0.25 V in all above experiments.

The preparation of F8T2‐SWCNT synaptic device arrays is detailed in Figure S1 (Supporting Information). **Figure** **1**a,b show the photographs of a 30 × 30 SWCNT TFT array and the magnified optical image of a single device in the array. The typical SEM image of the SWCNT network in Figure 1c demonstrates the uniform distribution of sc‐SWCNTs in the device channel. Figure 1d illustrates the structure of the bottom‐gate and bottom‐contact SWCNT TFT. The STEM‐HAADF image and the detailed EDS mapping of cross‐sectional F8T2‐SWCNT TFT are shown in Figure S2 (Supporting Information). The HRTEM image displays that the F8T2‐SWCNT film with a thickness of ≈5 nm is uniformly and continuously distributed in the channel (Figure 1e). The electrical properties of SWCNT TFTs are shown in Figure S1 (Supporting Information). Figure S3a (Supporting Information) displays the transfer curves of the 20 transistors selected randomly from flexible SWCNT TFT array. Clearly, the threshold voltage of the device is ≈0.7 V and the *I*_On_/*I*_Off_ ratio can reach 10^6^. Figure S3b–g (Supporting Information) illustrates the statistical plots of *I*_On_, *I*_Off_, Log (*I*_On_/*I*_Off_), *V*_th_, *SS*, and mobility extracted from the transfer curves of 150 randomly selected SWCNT TFTs on the PI substrates, where the statistical results prove the device performance uniformity in high‐performance SWCNT TFT arrays. Figure 1f is the optical UV–vis−NIR absorption spectra of the F8T2 film, the F8T2‐SWCNT film (the film consisting of sc‐SWCNTs sorted by F8T2), and the PFIID‐SWCNT film (the film consisting of sc‐SWCNTs sorted by PFIID). Compared with PFIID, the F8T2 film exhibits broadband absorption properties in the UV–vis region and tails in the NIR region, and thus the TFTs based on the F8T2‐SWCNT films shows photoresponsivity at NIR wavelengths, in contrast to the TFTs based on the PFIID‐SWCNT films, which shows almost no response at NIR wavelengths (Figure S4, Supporting Information). Figure 1h shows the transfer characteristics of the F8T2‐SWCNT TFT device in dark and exposed to different wavelengths of irradiation. Compared with the dark conditions, the transfer curves of TFT shift to the positive direction and on‐currents of the device increase under illumination of 365–940 nm light, indicating the excellent broadband response of the synaptic arrays from the UV to the NIR range. Noted that the transfer curves display more changes with a shorter wavelength light owing to larger photon absorption cross‐section. Figure S5 (Supporting Information) depicts the output characteristics of the F8T2‐SWCNT synapse transistor under dark and different wavelengths of light, where the *I*_DS_ increases with decreasing wavelength that is consistent with the Figure 1h. In addition, the storage characteristics of the device were preliminarily investigated. Figure 1i shows the typical transfer curve for the nonvolatile SWCNT memory device at a fixed drain voltage of −0.25 V. A significant shift in the threshold voltage and variable hysteresis window were induced when the gate voltage was scanned from ± 1 to ± 2.5 V (Between 2.5 and −2.5 V), and a large ∆*V*_th_ of ≈2.5 V can be obtained with a *V*_GS_ scan range of ± 2.5 V. That is to say, the size of the memory window (∆V) of the flexible SWCNT TFT depends on the scan range of *V*_GS_, which is in accordance with the basic electrical behavior of the memory device. Figure 1j shows the changes in *I*_DS_ of the F8T2‐SWCNT TFTs based memory device stimulated by the light with wavelengths ranging from 365 to 940 nm. The *I*_DS_ increases with the number of pulses increasing and the wavelength of the light decreasing. Moreover, the *I*_DS_ had no attenuate even for 250 s after the end of light excitation. Therefore, the F8T2‐SWCNT memory device exhibits required photosynaptic properties, good nonvolatile characteristics, and wide‐band photoresponses.

## 3 Optoelectronic Synaptic Properties of F8T2‐SWCNT Flexible Carbon Nanotube Transistors

**Figure 2:**
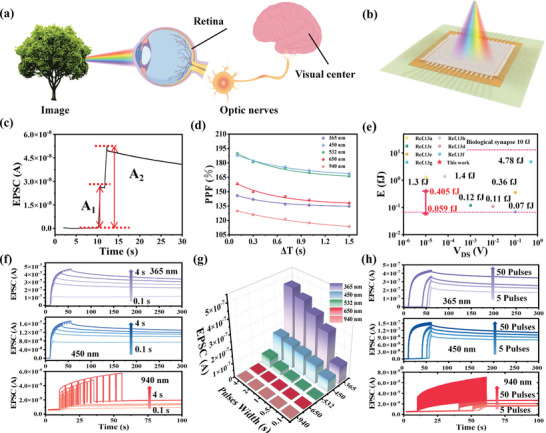
a) Schematic diagram of simulated human eye recognition of external objects. b) The broadband F8T2‐SWCNT synaptic phototransistor array. c) PPF behavior triggered by two consecutive photonic pulses (450 nm, 6 mW cm^−2^, 0.5 s) with the Δ*T* of 0.7 s. d) PPF index triggered by the photonic pulses (6 mW cm^−2^, 0.5 s) with the wavelengths of 365, 450, 532, 650, and 940 nm as a function of *ΔT*, respectively. e) The statistics chart of reported synaptic transistor power consumption. f) The EPSC curves and g) the maximum EPSC values with different pulses width at various light wavelength h) The EPSC curves with different number of pulses (6 mW cm^−2^, *V*_DS_ = −0.01 V).

The human perception of color‐image information from external environments starts the photoreceptors in the retina of the human eye and signals are then sent to the visual cortex, with the synaptic function, in the brain for processing and storage (As shown in **Figure** **2**a,b). Considering the ultrafast transmission, large bandwidth, and low interconnect energy loss of optical signals, our thin‐film transistors based on F8T2‐SWCNT active layer with the non‐volatile photoresponse to the UV–Vis−NIR band are very suitable optoelectronic synaptic devices. In a similar way to biological synapses, optical or electrical pulses can play the role of presynaptic pulses, depending on the mode of operation of the device. The postsynaptic current and synaptic weights are analogous to the drain current and channel conductance, respectively, and the change in synaptic weights is referred to as “synaptic plasticity”. In our device, 365 –940 nm light pulses are considered as presynaptic pulses and the *I*_DS_ is regarded as the excitatory postsynaptic current (EPSC). The paired pulse facilitation (PPF) behavior is an important manifestation of synaptic plasticity in biological synapses. In particular, when two continuous pulses with a sufficiently short time interval are input, the second pulse signal can induce a higher EPSC in the postsynaptic neuron compared with the first pulse signal. As shown in Figure 2c, PPF of the F8T2‐SWCNT based device was successfully simulated by using two identical light pulses (the pulse width of 0.5 s and the time interval of 0.7 s) and the EPSC was read at a *V*_DS_ of −0.01 V. PPF index can be defined by the following equation:

(1) $$\mathrm{PPF}\;\mathrm{index}=\frac{A_{2}}{A_{1}}\times 100\%$$

Additionally, the PPF index can also be fitted by a double exponential function:

(2) $$\mathrm{PPF}\;\mathrm{index}=1+C_{1}\cdot \exp\left(-\frac{\Delta T}{T_{1}}\right)+C_{2}\cdot \exp\left(-\frac{\Delta T}{T_{2}}\right)$$

where, *C*_1_ and *C*_2_ represent the original facilitation magnitude of slow and rapid phases, *τ*_1_ and *τ*_2_ represent the characteristic relaxation time of slow and rapid phases, respectively. In our case, PPF as a function of Δ*T* was studied by varying Δ*T* from 0.1 to 1.5 s (Figure 2d) and the experiment data can be highly fitted by Equation (2). It's worth noting that as the time interval (Δ*T*) between two light pulses increased, the PPF index induced by the light pulses decayed. In addition, the above experiments demonstrated that the devices excited by incident light between 365 and 940 nm can exhibit excellent synaptic plasticity, and the highest PPF index is 189.9% with the Δ*T* of 0.1 s and the light wavelength of 532 nm. The energy consumption (*E*) per light pulse can be defined as *E* = *V*_DS_ × *I*_EPSC_ × *t*, where *I_EPSC_* represents the maximum value of EPSC and *t* is light pulse width. When a fixed *V*_DS_ of −0.00001 V is applied, the *E* values are 59–405 aJ with the light pulse width from 0.02 to 0.1 s (Figure S6, Supporting Information). In particular, our synaptic devices have lower power consumption compared to previously reported synaptic transistors^[^ ^10^, ^13^ ^]^ (Figure 2e), which is more beneficial for developing neuromorphic computing.

As shown in Figure 2f,g and Figure S7a,b (Supporting Information) synaptic behaviors are demonstrated by applying ten pulses of different pulse widths at different wavelengths from 365 to 940 nm (6 mW cm^−2^, 0.5 s), confirming that the transistor has a broadband synaptic response from UV to NIR range light. Furthermore, among five different wavelengths of light pulses, the largest EPSC (up to 4.72 × 10^−7^ A) could be acquired by stimulated by 365 nm wavelengths of monochromatic light, while the light pulses with the wavelength of 940 nm resulted in the smallest EPSC (up to 5.8 × 10^−9^ A) for the same light pulses train, which were in agreement with the results above. The synaptic characteristic can also be well regulated by adjusting the duration of the light pulses. For instance, with the light pulse duration varying from 0.1 to 4 s, the maximum value of EPSC rises from 1.26 × 10^−7^ to 1.72 × 10^−7^ A with the light wavelength of 450 nm and *V*_DS_ of −0.01 V. Significantly, the EPSC still maintains >80% of the peak current after 250 s of applying the light pulses with a wavelength of <450 nm, which is due to the superior charge trapping of AlO_x_ grown by ALD at low temperatures, compared with those grown at a typical temperature ≈250 °C (Figure S8, Supporting Information). Similarly, as depicted in Figure 2h and Figure S7c–e (Supporting Information), the EPSC enhanced with the increase of the number of the light pulses with the different wavelengths. To verify the homogeneity of the array, we applied five light pulses of different wavelengths (6 mW cm^−2^, 0.5 s). 20 devices were randomly selected from the array and their EPSC curves were plotted after photostimulation (Figure S9, Supporting Information), and the peak EPSC values of 50 randomly selected devices were extracted and plotted as histograms (Figure S10, Supporting Information). The above experimental results demonstrate the homogeneity of our proposed array. Subsequently, we tested the stability of the F8T2‐SWCNT TFT by testing the response to 5 light pulses at a wavelength of 450 nm (6 mW cm^−2^, 0.5 s) at different times. As shown in Figure S11 (Supporting Information), the photocurrent of the F8T2‐SWCNT TFT had no obvious degradation over the 45 day test period, which proves the excellent stability of our proposed device. These results demonstrate the broadband synaptic properties of the F8T2‐SWCNT synaptic transistors. In addition, the photosynaptic transistor exhibits a fast response time of 80 ms, a high responsivity of up to 487.9 A W^−1^ and the good mechanical flexibility (Figures S12 and S13, Supporting Information).

## 4 The Multistate Storage Capacity of Light Programmed Flexible F8T2‐SWCNT Transistors

**Figure 3:**
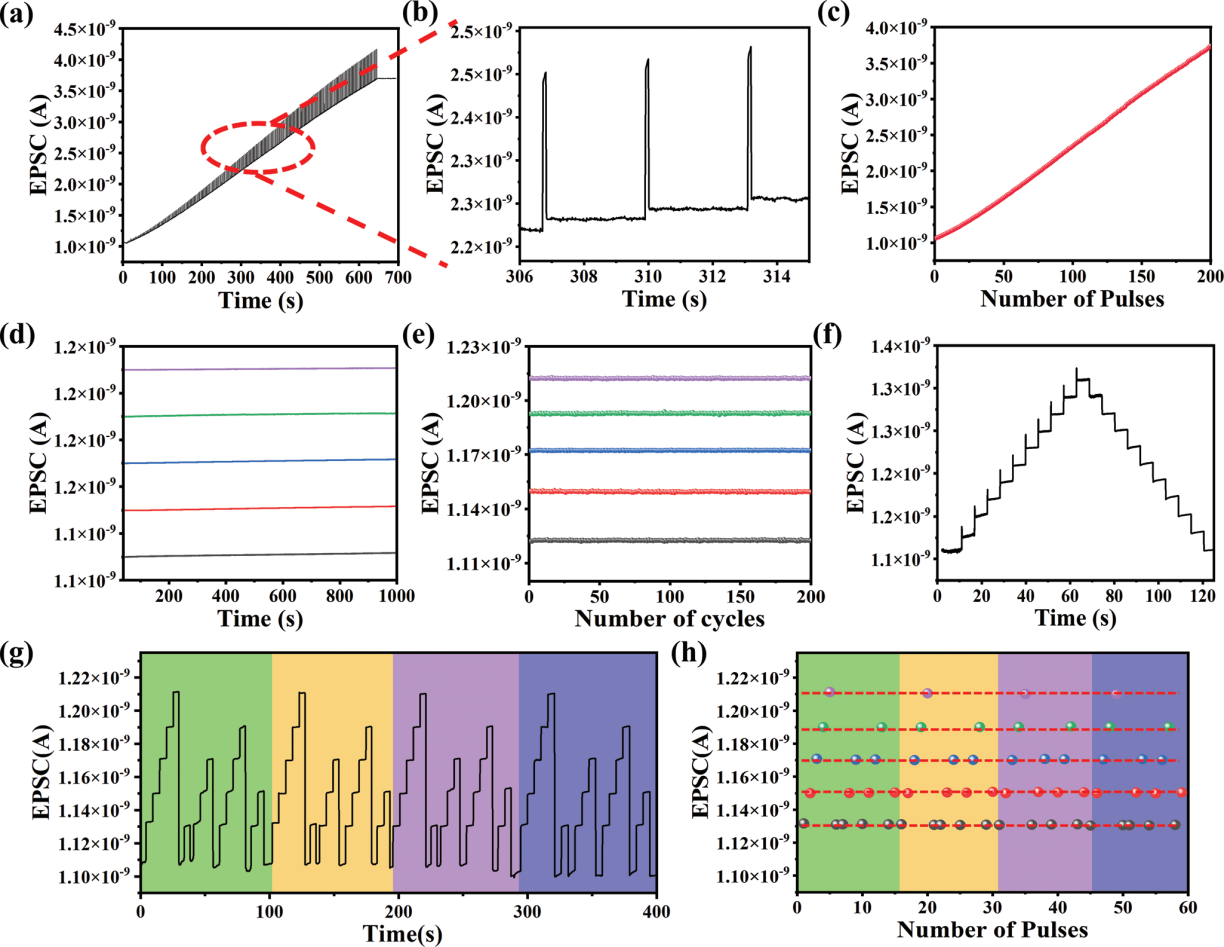
Multistate storage capacity of F8T2‐SWCNT synaptic transistor. a) Dynamic behavior of the F8T2‐SWCNT synaptic transistor under light illumination (450 nm, 6 mW cm^−2^, 0.04 s). b) Magnified view of 5 current states from a). c) Plot of EPSC with respect to the number of applied light pulses. d) Retention of the magnified 5 states measured up to 1000 s. e) Cycling tests of the F8T2‐SWCNT synaptic transistor under photo illumination at various current states. f) The long‐term potentiation and the long‐term depression induced by the stimulation trains of 10 consecutive light programming pulses (6 mW cm^−2^, 0.1 s) and 10 gradient electrical erasing pulses. g) The flexible switching of five different current states. h) Cycle stability of flexible switching.

Thanks to the excellent non‐volatile photosynaptic properties, the F8T2‐SWCNT synaptic transistors exhibit the capacity of multibit storage under the optical programming and electrical erase operation. In order to further study the storage capacity of light programmed F8T2‐SWCNT transistors, the dynamic behavior of the synaptic transistor was recorded under 650 nm optical and electrical pulse sequence. It is important to emphasize that the devices used for multilevel storage testing were passivated with polyvinyl pyrrolidone (PVP) to reduce the effects of airborne water and oxygen, the specific experimental methods refer to previous studies.^[^ ^14^ ^]^ As shown in **Figure** **3**a, the intensity and width of each light pulse are set as 6 mW cm^−2^ and 0.04 s, respectively, and the interval of 3 s between each pulse is used to assess the retention ability of EPSC. Specifically, 200 states can be obtained before current saturation, suggesting excellent multibit storage performance of the F8T2‐SWCNT transistors. As shown in Figure 3b, an enlarged view of the several specific storage states from Figure 3a is provided, each of which is well separated and maintained with ignorable current degradation during the pulse interval. It's remarkable that the current difference of only 0.02 nA between the two states is significantly greater than any noise or fluctuation during the test. This proves that the two states can be effectively separated and distinguished to ensure the fidelity of data. The Δ*I*_DS_ rises linearly with an increasing number of pulses as indicated by the scatter plot (Figure 3c) and the high storage capacity of the synaptic device is demonstrated. The first five of these optically programmed pulses are selected for further study, and their current values are defined as state 1–5, respectively. Notably, the five states can be maintained for >10^3^ s as illustrated in Figure 3d, indicating that each state is highly nonvolatile. Subsequently, the cycle stability of writing‐erasing process was tested and the applied stimulation sequence consists of 5 consecutive light pulses followed by erasing with a −1.35 V electrical pulse with the pulse duration of 0.1 s in every cycle. In Figure 3e, the device showed good stability over 200 cycles with the current value changed by <5% (cf: devices without PVP encapsulation exhibit poor stability as shown in Figure S14, Supporting Information). To verify the homogeneity, we tested the retention as well as the cyclic stability of 10 randomly selected devices from the array. As shown in Figures S15 and S16 (Supporting Information) all tested devices exhibit a hold time of >10^3^ s as well as good cyclic stability, demonstrating the good homogeneity of our proposed array.

Multilevel storage and flexible switching of different storage states are especially important for AI multisensory systems that need to receive multiple stimulus signals from different objects.^[^ ^15^ ^]^ Ten storage states were written by 650 nm light pulses in Figure 3f. Then the gradient negative electrical pulse train (amplitude increases from −0.15 to −1.35 V) was used to realize the symmetry erasing. The above results demonstrate the long‐term photonic potentiation and the long‐term electric depression in F8T2‐SWCNT TFT device. As shown in Figure 3g, different numbers of optical pulses (6 mW cm^−2^, 0.04 s, 650 nm) and the corresponding electrical pulses (0.1 s) can be applied to achieve flexible switching of different storage states, and successive 4 operation cycles were obtained. The test route for one operation cycle consisting of 10 switching states is as follows. First, 5 light pulses were applied to switch the device from the initial state (“state 0”) to the “state 5”. It should be noted that “state 5” was obtained after experiencing 4 states (from “state 1” to “state 4”). Next, the “state 5” was erased by a voltage pulse with the value of −0.75 V and the duration of 0.1 s, which returned the device to the “state 0”. Then, the “state 1” was written through 1 light pulse to realize the switch from “state 0” to “state 1”, followed by an electric pulse to erase the “state 1” to “state 0”. Subsequently, the “state 3”, “state 4” and “state 2” were programmed and erased continuously using similar schemes. As a note, the value of the erasing voltage pulse is −0.15 and −0.3 V, −0.45 and −0.6 V for “state 1”, “state 2”, “state 3”, and “state 4”, respectively. Figure S17 (Supporting Information) exhibits a schematic diagram of the operating process used for multiple memorizing steps. As shown in Figure 3h, the storage state currents were almost not shifted after a number of corresponding switching cycles, and the currents of “state 1”, “state 2”, “state 3”, “state 4”, and “state 5” are mainly gathered in 1.13 × 10^−9^, 1.15 × 10^−9^, 1.17 × 10^−9^, 1.19 × 10^−9^, and 1.21 × 10^−9^ A, respectively, which prove the switching reliability of various storage states. The above experimental results show that the F8T2‐SWCNT transistors can achieve flexible switching of different storage states by optical programming and electrical erasure, which provides potential possibilities for the realization of artificial vision systems adapted to multiple complex environments. The results of our study are summarized in Table S1 (Supporting Information) and compared with the critical characteristics (such as recognizable wavelength, power consumption per pulse, as well as the number of multistate) of recently reported photoelectric synaptic devices with multistate memories. It is evident that our F8T2‐SWCNT TFT possess low operating voltage (from −1.5 to 1 V), large *I*_On_/*I*_Off_ ratio (>10^6^), broadband photoresponse (from 365 to 940 nm), and excellent multilevel storage performance (200 conductance states).

## 5 Working Mechanism

**Figure 4:**
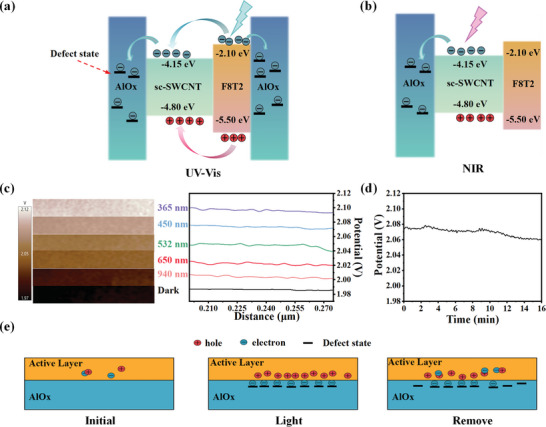
Schematic band diagram of SWCNT/F8T2 and the charge transfer between SWCNT, F8T2, and AlO_x_ under a) UV–vis and b) NIR light illumination. c) Surface potential of the SWCNT film under dark and the illumination with different wavelengths from 365 to 940 nm, respectively. d) Surface potentials of SWCNT at 0 and 16 min after stimulation with 450 nm light. e) Schematic illustration of the charge trapping effect at the interface between the active layer and the dielectric layer of an artificial synaptic TFT when irradiated by the pulsed light.

During the photo programming, the transfer curve shifted positively, suggesting that holes are the main conduction carriers. Note that the highest occupied molecular orbital (HOMO) and lowest unoccupied molecular orbital (LUMO) energy levels of F8T2 are −5.50 and −2.10 eV.^[^ ^16^ ^]^ When the incident photon energy is larger than the bandgap of F8T2, holes are transferred to SWCNTs while electrons are directly trapped by AlO_x_ or indirectly move to AlO_x_, leading to the separation of electrons and holes (**Figure** **4**a). When the incident photon is with a lower energy (smaller than F8T2 bandgap), the excitons produced in SWCNTs will be separated by transferring electronics directly to AlO_x_, as illustrated in Figure 4b. In addition, SWCNT absorbs water and oxygen from the air, and the transfer of electrons from SWCNT to redox pairs leads to an increase in the concentration of holes in SWCNT, which increases the EPSC value and affects the stability of the SWCNT device in air. The PVP passivation method described above isolates some of the effects of water and oxygen in air.

To investigate the reconfiguration of charge carriers associated with tunable synaptic plasticity in the transistor, KPFM was used to characterize the surface potential of sc‐SWCNT as shown in Figure 4c. When a light pulse with a wavelength of 365 nm was applied, the surface potential of the sc‐SWCNT film increased from the 1.988 V (original state) to 2.089 V. Moreover, the difference between the surface potential values from sc‐SWCNT films under the light condition and dark condition gradually increased with decreasing the light wavelength, which corresponded to the wavelength‐dependent broadband synaptic behavior. It should be noted that the surface potential of the SWCNT film decreased by only 20% at the 16 min after completing irradiation by the 450 nm light pulses compared with that at the beginning (Figure 4d), which corresponds to the slow decay process of the postsynaptic current after photostimulation. Correspondingly, Figure S18 (Supporting Information) shows the KPFM image of the SWCNT film after the light stimulation at 0 and 16 min. The memory characteristic of SWCNT phototransistor is also relevant to the charge trapping effect of AlO_x_ (Figure 4e). Initially, the transistor channel has only a few free carriers in the absence of light. When the device is stimulated by light, some of photogenerated electrons are trapped by the defect states of AlO_x_, and most of them are still kept in the AlO_x_ dielectrics or the interfaces after light pulse stimulation, which is due to the good charge trapping ability of the AlO_x_ dielectric layer grown by ALD at low‐temperature.^[^ ^4^ ^b,^ ^17^ ^]^ It needs to be emphasized that because F8T2 itself and the F8T2/SWCNT interface provide sufficient charge trapping states, the recombination of photogenerated carriers can be suppressed effectively, which is also benefit for SWCNTs memory device applications. Therefore, the SWCNT photoelectric transistor with good charge retention property exhibits the great potential to be applied as a photoresponsive nonvolatile memory device.

## 6 The Simulation of the Visual Persistence of the Human Eye

**Figure 5:**
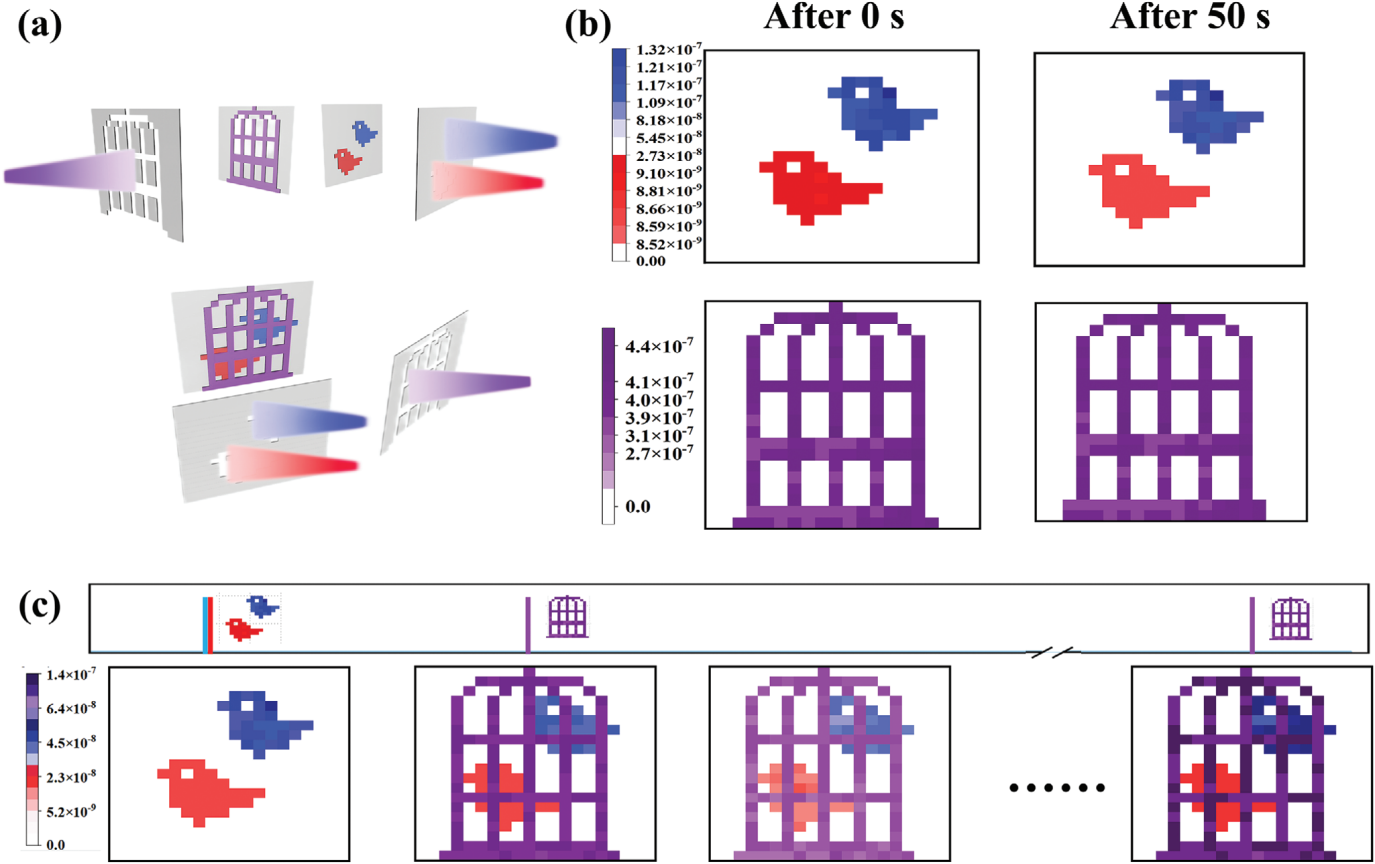
a) Schematic of simulated visual persistence behavior based on a 20 × 20 pixels array of photosynaptic transistors. b) “Bird” patterns and “Birdcage” patterns of channel conductance states after 50 s of decay following stimulation with a photon pulse sequence of 10 pulses. c) The process of simulating visual persistence behavior by alternating visual corresponding wavelengths of light.

Visual persistence behavior is the key to achieving continuous dynamic image recognition in the computer vision that has become one of the research hotspots in artificial intelligence technology in recent years.^[^ ^18^ ^]^ Here, the famous “caged bird” experiment was executed to simulate visual persistence phenomenon in our F8T2‐SWCNT photosynaptic arrays with 20 × 20 pixels by studying the visual memory of the devices that can be represented by the EPSC.As shown in **Figure** **5**a, the photoresponse of the synaptic devices array was investigated by using light pulse stimulation with different wavelengths (365, 450, and 650 nm). As shown in Figure 5a,b, the purple‐patterned “birdcage”, blue‐patterned “birds”, and red‐patterned “birds” could be excited by 10 patterned incident light pulses (6 mW cm^−2^, 0.5 s) with the wavelength of 365, 450, and 650 nm, respectively, and the color intensity of the pattern represented different levels of memory. The patterns of different colors can remain clear for 50 s after the light pulses were removed, suggesting that the synaptic arrays have excellent visual memory effects. Subsequently, visual persistence was simulated by alternately applying light of corresponding wavelengths (Figure 5c). First, the 450 and 650 nm light pulses were applied respectively. The array exhibited a distinct image of the “birds”. After 10 s, the 365 nm light was applied to obtain “cage” pattern on the array. Due to the excellent non‐volatile characteristics of the F8T2‐SWCNT devices, the “bird” pattern was still clearly retained, thus realizing the overlap of the bird and cage patterns—the bird “flying” into the cage. The pattern in the array became blurred with time, and again after 10 s, the current in the “bird” is only 70% of the peak EPSC value. The above was then repeated and as the number of cycles increased, the pattern became clearer.

## 7 Simulation of Neuromorphic Vision System Based on SNN Algorithm

**Figure 6:**
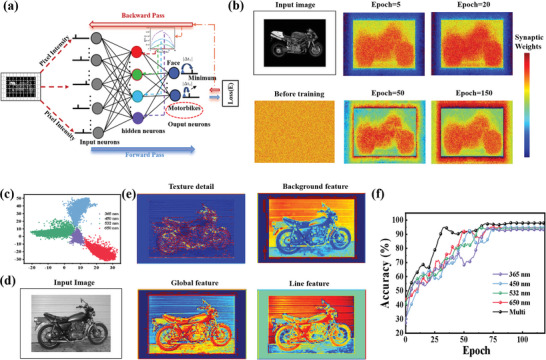
a) Schematic diagram of schematic illustration of constructed MLP‐based SNN. b) Mapping images of synaptic weights. c) The feature maps learned by neurons in different wavelengths. d) Input image. e) The feature distribution map of the light pulses with different wavelengths. f) The recognition accuracy evolution of the light pulses with different wavelengths.

This paper proposes a two‐layer perception based on SNN algorithm, consisting of 160 × 250 input neurons, 400 hidden neurons, and 2 output neurons, for the Caltech 101^[^ ^19^ ^]^ dataset (160 × 250 pixels) recognition task, to validate the exceptional synaptic performance of our devices within the neuromorphic system. An overview of the SNN algorithm is shown in **Figure** **6**a. During the inference, the input image's real‐valued pixel intensities are converted to the information as the latency to the first spike of the corresponding spike train.^[^ ^20^ ^]^ In the hidden layer, each LIF neuron integrates weighted spikes and generates an output spike once the membrane potential surpasses the threshold potential. The LIF neuron works as follows:

(3) $$u\left(t+1\right)=\tau u\left(t\right)+W\cdot x\left(t\right)$$

(4) $$\left\{\begin{array}{c} O\left(t+1\right)=\theta \left(u\left(t+1\right)-V_{\mathrm{th}}\right)\\ u\left(t+1\right)=u\left(t+1\right)\left[1-O\left(t+1\right)\right] \end{array}\right.$$

where, *u(t)* is the membrane potential of the neuron at time *t*, *x(t)* is the input spikes, *τ* is the constant of leakage, *W* is the synaptic weight, *O(t)* is the output spike, *V*_th_ is the threshold potential.

After completing the forward process, the input image's category is determined by the output neuron that generates the earliest spike. The weights in the neural network are updated using backward propagation, which requires obtaining the derivative of the loss function with respect to each weight. The gradient is then propagated backward to the hidden layers by employing the chain rule, as shown in the following equation.

(5) $$\frac{\partial E}{\partial w^{l}}=\frac{\partial E}{\partial t^{l}}\;\frac{\partial t^{l}}{\partial w^{l}}$$

Before using the LTP/LTD characteristic curves to compute the derivative between post‐synaptic firing time and the weight, we design a transformation function F(.) to make the curves fit our SNN algorithm better.

(6) $$f\left(x\right)=F(g\left(x\right)=\left\{\begin{array}{c} g\left(-\left(x+T_{\max}\right)\right)/V_{\mathrm{DS}},x+T_{\max}<0\\ -g\left(-\left(x+T_{\max}\right)\right)/V_{\mathrm{DS}},x+T_{\max}>0 \end{array}\right.$$

where, f(x) is the LTP/D curve after transformation, g(x) is the original LTP/D curve, *T*_MAX_ is the maximum number of pulses, and *V*_DS_ is the bias voltage of the device.

According to the definition of spike‐time‐dependent plasticity,^[^ ^21^ ^]^ strengthening occurs when the presynaptic spike precedes the postsynaptic spike, and thus we use the LTP curve to increase the weight. And if the pre‐synaptic spike occurs after the post‐synaptic one, the connection becomes ineffectual, and thus we use the LTD curve to reduce the weight.

(7) $$\frac{\partial t^{l}}{\partial w^{l}}=\left\{\begin{array}{c} f_{\mathrm{LTP}}\left(t_{\mathrm{post}}-t_{\mathrm{pre}}\right),t_{\mathrm{post}}>t_{\mathrm{pre}}\\ f_{\mathrm{LTD}}\left(t_{\mathrm{post}}-t_{\mathrm{pre}}\right),t_{\mathrm{post}}<t_{\mathrm{pre}} \end{array}\right.$$

where, *t*_pre_ and *t*_post_ represent the recorded spike times of a pre‐ and post‐neuron pair. *f*_LTP_ and *f*_LTD_ are the LTP/D characteristic of the f(x).

In order to validate the efficacy of the hardware‐based SNN simulation algorithm, the mapping images of the 160 × 250 synaptic weights connected to the input image are plotted during training in Figure 6b. The visualization results show that the network attention is focused on the key feature parts of the target, which illustrates the effectiveness of the feature extraction ability of the SNN model in this paper. When stimulated by light pulses of different frequencies, our synaptic devices get different LTP/D curve responses. It should be emphasized that the LTP/D curves we obtained have good linearity, symmetry, and cyclic stability (Figures S19 and S20, Supporting Information). Thus, as shown in Figure 6a, we divide the 400 neurons of the hidden layer into four groups equally, and apply the LTP/D curves of light pulses with wavelengths 365, 450, 532, and 650 nm for weight update, respectively.

In order to explain the role of the multi‐frequency curve in the algorithm, we visualized the feature map of these four types of neurons in the hidden layer, as shown in Figure 6d. The initialization information of the algorithm simulation is listed in Table S2 (Supporting Information). From the visualization results, we can see that the four types of neurons pay attention to different features of the input image, respectively are texture detail, background, global information, and line feature. Figure 6e,f illustrate that the feature distributions learned by neurons in different bands are clearly bounded, indicating that they have learned knowledge of different feature spaces, and thus the recognition accuracy of the multi‐band algorithm is the highest, reaching 97.92%. However, the single frequency curve algorithm has poor stability and low recognition accuracy. Then, to evaluate the effectiveness of the learning algorithm that we have proposed, we evaluated the test accuracy of our method on the Modified National Institute of Standards and Technology database (MNIST). The specific results are shown in Figures S21 and S22 (Supporting Information).

## 8 Conclusion

In this article, we present a novel SWCNT photoelectric synaptic transistor array with broadband photoresponse and multilevel storage by using a simple preparation method. The photosensitive polymer F8T2 was chosen to wrap the carbon nanotubes, and the AlO_x_ grown at 120 °C was used as a dielectric layer to improve the ability to trap charge. Under the incident light from the UV (365 nm) to NIR (940 nm) wavelength range, the F8T2‐SWCNT TFT device exhibits excellent synaptic performance with a single pulse power consumption as low as 59 aJ. Due to the AlO_x_ grown by ALD at low‐temperature and polymer wrapping, the device can obtain 200 well‐separated storage states with excellent non‐volatility. Furthermore, a SNN algorithm is proposed for neuromorphic devices to realize the recognition task of Caltech 101 dataset. More importantly, for the first time, we implement the algorithm to extract different features from the images by different frequency curves, and significantly enhance the accuracy and stability of the recognition task.

## 9 Experimental Section

### Materials

Arc discharge SWCNTs (P2, the diameter of SWCNTs from 1.2 to 1.6 nm) were purchased from Carbon Solution. (9,9‐Dioctylfluorene‐co‐bithiophene) (F8T2) (Mw/Mn = 21 000/12 000) was purchased from Shenzhen (China) Derthon Optoelectronic Materials Science & Technology. All products were directly used without further purification.

### Preparation of sc‐SWCNT Inks

To obtain sorted sc‐SWCNT solution, 4 mg of P2 was dispersed in 5 mL xylene with 10 mg F8T2 via probe‐ultrasonication (Sonics & Materials, model: VCX 13060 W) for 30 min. Then, the homogenized dispersion was centrifuged at 15 000 and 20 800 rpm for 1 h, respectively, to remove metallic species and big bundles. The supernatant (the resulting sc‐SWCNT inks) was drawn out from the centrifuge tubes and used to fabricate SWCNT TFTs without any other purification.

### Device Fabrication

The SWCNT TFTs were manufactured on PI substrates with a bottom‐gate/bottom‐contact structure. Gate electrodes (120 nm Al) were fabricated by standard photolithography, thermal vapor deposition, and lift‐off processes. A 15 nm AlO_x_ dielectric layer was then deposited on the gate electrodes by atomic layer deposition (ALD) technique (trimethyl aluminum and water as precursors, 120 °C). Next, source and drain electrodes were formatted on the dielectric layer by the aforementioned method with different metal materials (Ag). Subsequently, sc‐SWCNT solution was printed onto the surface of dielectric layer that was treated by UV‐ozone for 3 min to improve the adhesion ability between sc‐SWCNTs and AlO_x_. After that, the devices were annealed at 120 °C for 3 min, followed by washing with xylene for three times to remove the excess polymers. Finally, the devices were annealed at 120 °C for 3 min to remove residual solvents.

### Characterization and Measurements

The absorbance spectra of F8T2‐sorted sc‐SWCNT inks were acquired using a UV–Vis−NIR spectrophotometer (Lambda 750, Perkin Elmer). The surface morphologies of the sc‐SWCNT networks were conducted by Scanning electron microscopy (SEM, Nova NanoSEM450). Preparation of the device interface structure was used by a focused ion beam (FIB) (FEI Scios) and the detailed EDX element analysis were executed through STEM (Tecnai G2 F20 S‐Twin). The in situ surface potential measurements were performed in ambient conditions at room temperature on AFM (Asylum Research MFP‐3D). Electrical characteristics of all F8T2‐SWCNT TFTs devices were recorded using Keithley 2636B, Keithley 4200, or Keithley 1500 electrometer system at room temperature. The equations of subthreshold swing (*SS*): $SS=\frac{dV_{\mathrm{GS}}}{d(\log I_{\mathrm{DS}})}$. The field effect mobilities were calculated using the standard linear regime: $\mu =10^{4}\times \frac{dI_{\mathrm{DS}}}{dV_{\mathrm{GS}}}\times \frac{L}{W}\times \frac{1}{C_{\mathrm{ox}}V_{\mathrm{DS}}}$. Here, *C*_ox_ is the Ion‐gel dielectric capacitance per unit area, which was 0.06 µF cm^−2^ at 100 kHz (Measured by a Keithley 4200 electrometer system), as well as the L and W were the channel length (3 µm) and width (250 µm), respectively.

## Conflict of Interest

The authors declare no conflict of interest.

## Author Contributions

N.S., Y.J., M.L, and F.Z. contributed equally to this work. N.S., M.L., and F.Z. performed experiments, wrote the original draft, and reviewed and edited the final manuscript. Y.J. performed simulation, wrote the original draft, and reviewed and edited the final manuscript. S.S., J.L., and Z.L. wrote the original draft, and reviewed and edited the final manuscript. J.W. performed conceptualization, wrote the original draft, and reviewed and edited the final manuscript. L.L. performed validation, acquired funding, wrote the original draft, and reviewed and edited the final manuscript. J.Z. performed validation, conceptualization, project administration, funding acquisition, wrote the original draft, and reviewed and edited the final manuscript.

## Supporting information

Supporting Information

## Data Availability

The data that support the findings of this study are available from the corresponding author upon reasonable request.
