# Supplementary material for: Photoprogrammed Multifunctional Optoelectronic Synaptic Transistor Arrays Based on Photosensitive Polymer‐Sorted Semiconducting Single‐Walled Carbon Nanotubes for Image Recognition
Source: Adv Sci (Weinh). 2024 Jun 3;11(29):2401794. doi: 10.1002/advs.202401794 (PMC11304235; doi:10.1002/advs.202401794)
Supplement: Supplementary file 1 — Supporting Information [file ADVS-11-2401794-s001.pdf]

ADVANCED SCIENCE
Open Access

# Supporting Information

for *Adv. Sci.*, DOI 10.1002/advs.202401794

Photoprogrammed Multifunctional Optoelectronic Synaptic Transistor Arrays Based on Photosensitive Polymer-Sorted Semiconducting Single-Walled Carbon Nanotubes for Image Recognition

*Nianzi Sui, Yixi Ji, Min Li, Fanyuan Zheng, Shuangshuang Shao, Jiaqi Li, Zhaoxin Liu, Jinjian Wu*, Jianwen Zhao* and Lain-Jong Li**

## Supporting Information

**Photoprogrammed multifunctional optoelectronic synaptic transistor arrays based on photosensitive polymer-sorted semiconducting single-walled carbon nanotubes for image recognition**

Nianzi Sui[1,2#], Yixi Ji[3#], Min Li[1,2#], Fanyuan Zheng[4#], Shuangshuang Shao[1,2], Jiaqi Li[1,2], Zhaoxin Liu[3], Jinjian Wu[3]*, Jianwen Zhao[1,2]*, Lain-Jong Li[4]*

[1]School of Nano-Tech and Nano-Bionics, University of Science and Technology of China, No. 398 Ruoshui Road, Suzhou Industrial Park, Suzhou, Jiangsu Province, 215123, PR China

[2]Division of Nanodevices and Related Nanomaterials, Suzhou Institute of Nano-Tech and Nano-Bionics, Chinese Academy of Sciences, No. 398 Ruoshui Road, Suzhou Industrial Park, Suzhou, Jiangsu Province, 215123, PR China

[3]School of Artificial Intelligence, Xidian University, Xi'an, 710071, PR China.

[4]Department of Mechanical Engineering, The University of Hong Kong, Pokfulam Road, Hong Kong, PR China.

* Corresponding authors.

Email: J.W. Zhao (jwzhao2011@sinano.ac.cn); J.J Wu (jinjian.wu@mail.xidian.edu.cn); L.J. Li (lanceli1@hku.hk)

**This file includes:**

Figure S1 to S22;

Tables S1 and S2

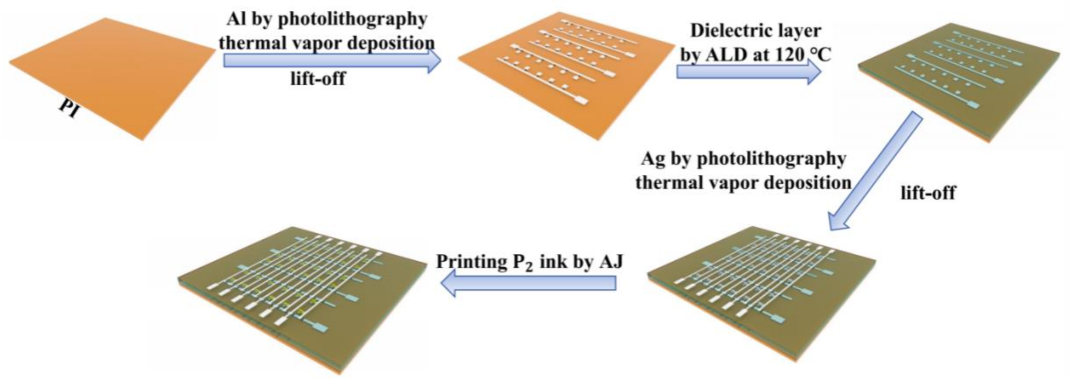


**Figure S1.** Schematic diagram of the manufacturing process of F8T2 SWCNT TFTs.

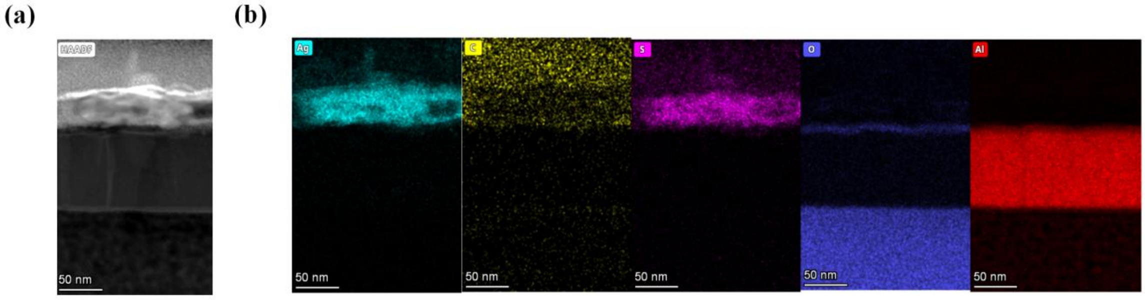


**Figure S2.** (a) STEM-HAADF image of cross-sectional F8T2-SWCNT TFT. (b) STEM-EDS mapping of (a).

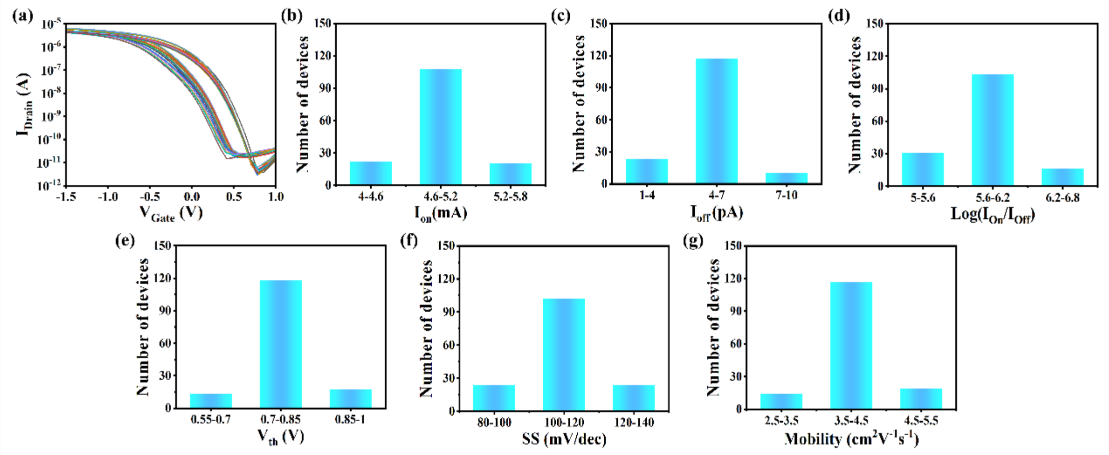


**Figure S3.** (a) The transfer curves of the SWCNT TFTs. The statistical distribution histograms of electrical properties for 150 devices randomly chosen from the TFT arrays (30 × 30). (b) $I_{On}$, (c) $I_{Off}$, (d) Log ($I_{On}/I_{Off}$), (e) $V_{th}$, (f) $SS$ and (g) *Mobility*.

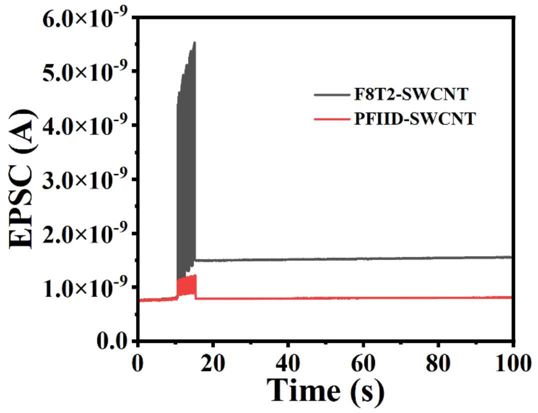


**Figure S4.** The comparison of the response of the TFT based on F8T2-SWCNT film and TFTs based on PFIID-SWCNT film to 940 nm wavelength light pulse stimulation.

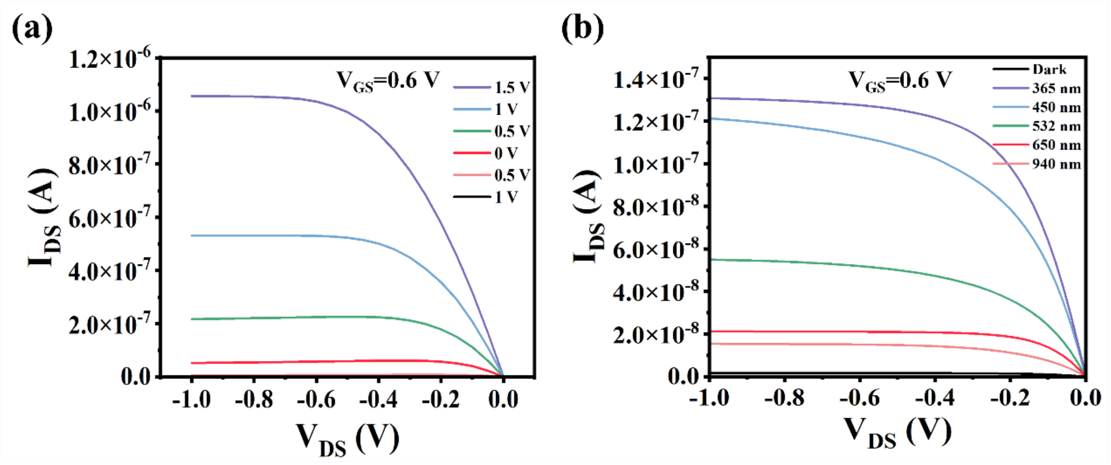


**Figure S5.** Output characteristic curves of the broadband optoelectronic synaptic transistor under (a) dark and (b) light illumination conditions (from 365 nm to 940 nm) ($V_{GS}$ = 0.6 V).

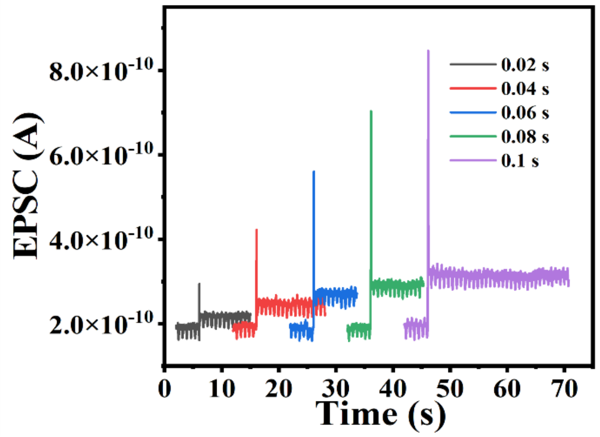


**Figure S6.** EPSCs under different pulses width at $V_{DS}$ of -0.00001 V.

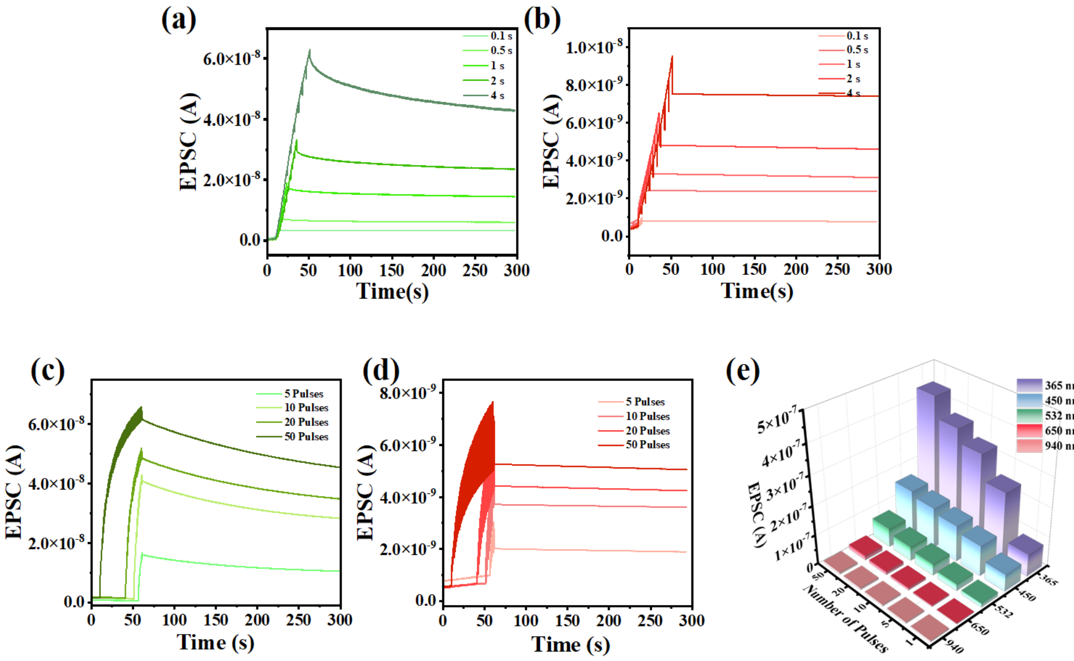


**Figure S7.** The EPSC curves with different pulses width at (a) 532 nm and (b) 650 nm. The EPSC curves with different number of pulses at (c) 532 nm and (d) 650 nm. (e)The maximum values of EPSC with the number of light pulses.

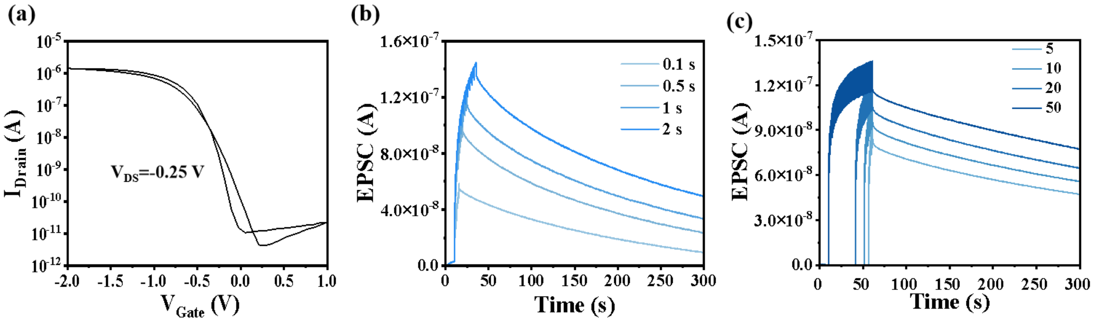


**Figure S8.** The transfer characteristics and light responses of the SWCNT TFT with the dielectric layer of $AlO_x$ grown by ALD at 250 °C. (a) The transfer curve. EPSCs triggered by 450 nm light (6 mW $cm^{-2}$) with (b) different pulses width and (c) different pulses number at $V_{DS}$ of -0.01 V.

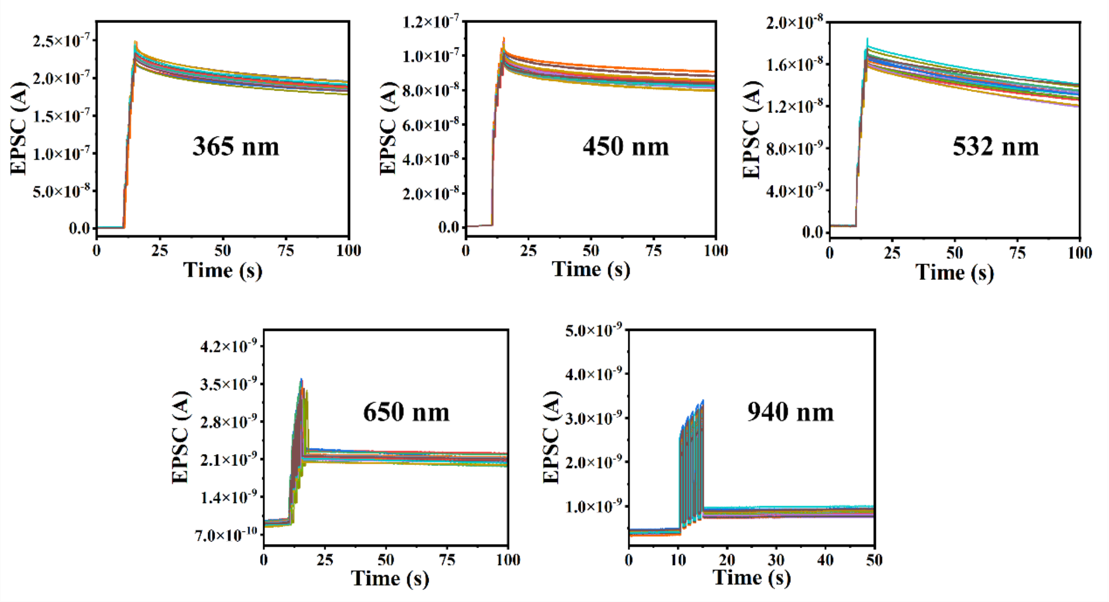


**Figure S9**. The EPSC curves of 20 randomly selected devices from the TFT array (30×30) after stimulation with light pulses (6 mW cm$^{-2}$, the pulse width = 0.5 s) of different wavelengths (from 365 to 940 nm).

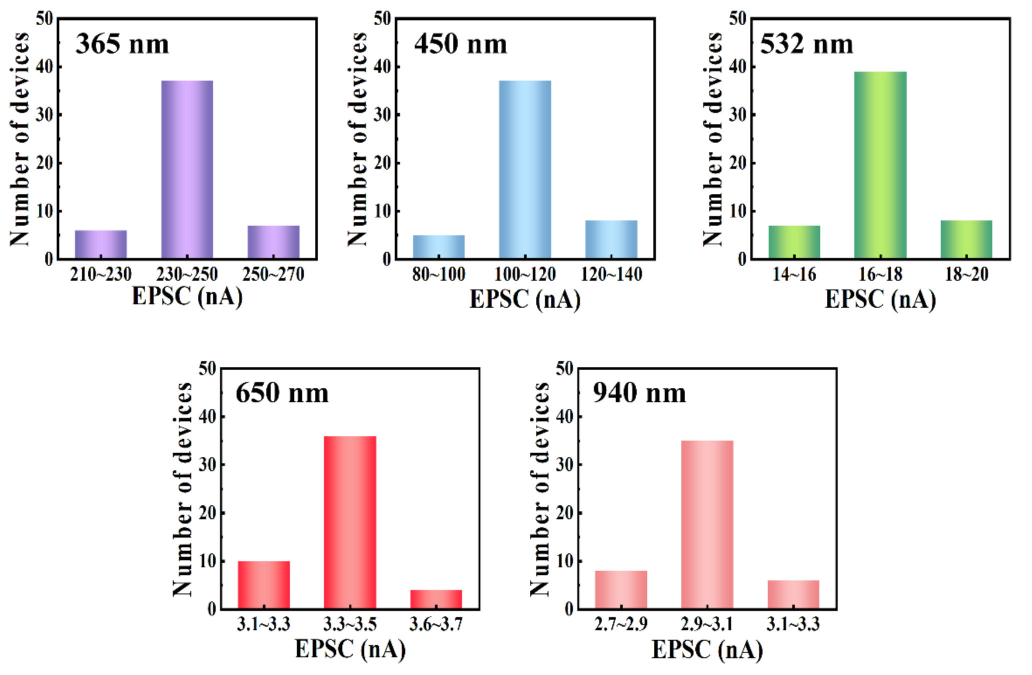


**Figure S10.** Distribution histogram of EPSC peaks of 50 randomly selected devices from the TFT array (30 × 30) after stimulation with light pulses (6 mW cm$^{-2}$, 0.5 s) of different wavelengths (from 365 to 940 nm).

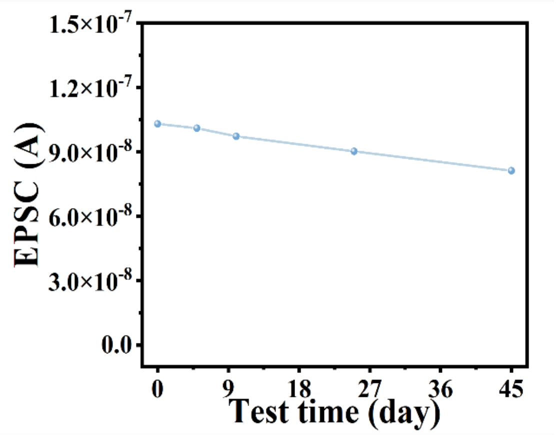


**Figure S11.** The EPSC peaks measured during the period of 45 days.

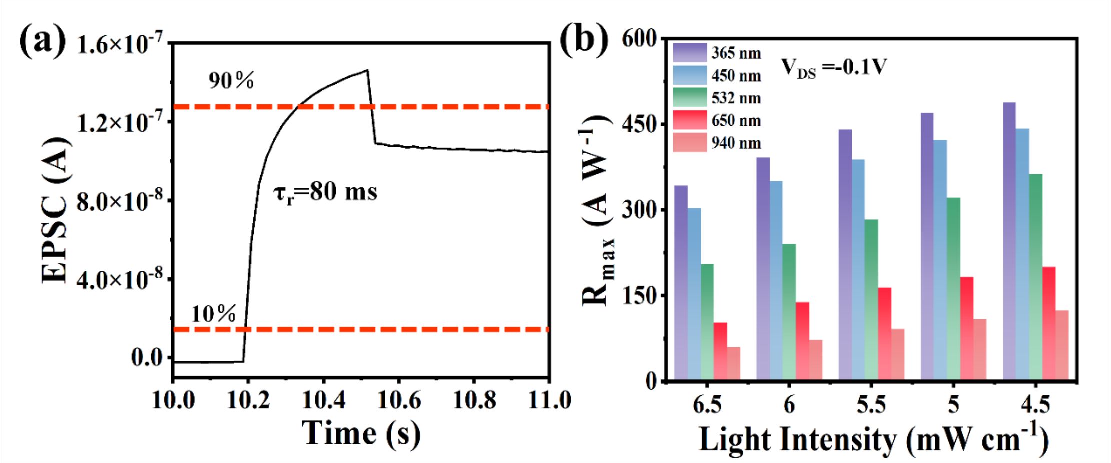


**Figure S12.** (a) The light response speed to 450 nm light stimulation (10 mW cm$^{-2}$, 0.3 s). (b) The photoresponsivity under different light intensities and wavelengths at $V_{DS}$ of -0.1 V.

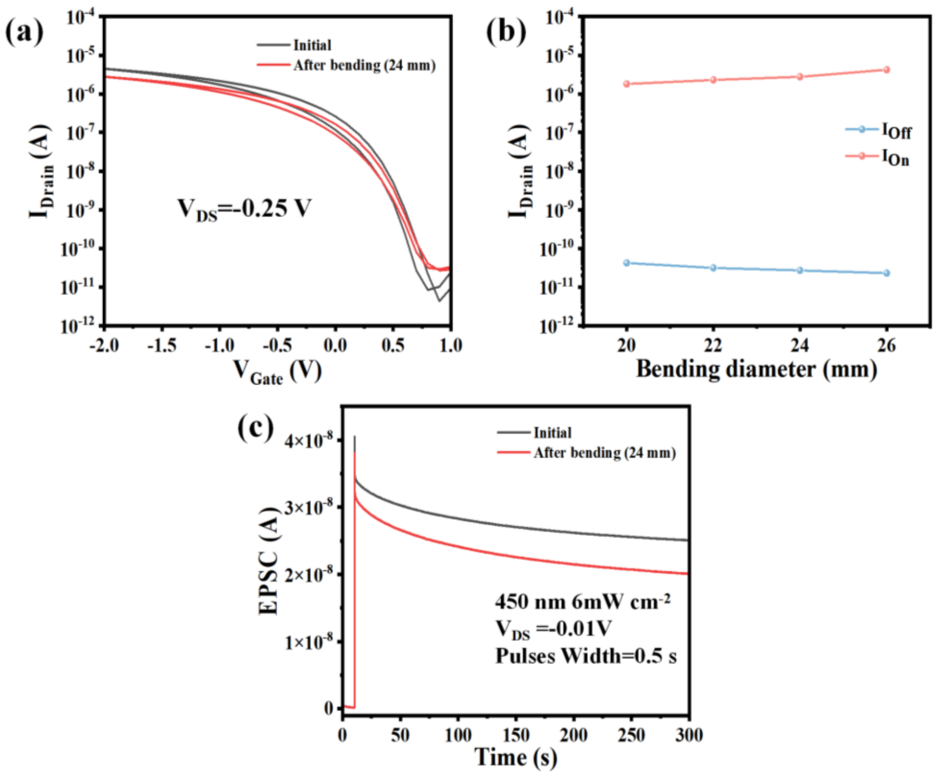


**Figure S13.** (a) Transfer curves before and after bending ($V_{DS}$ = -0.25 V). (b) on-state current ($I_{On}$), off-state current ($I_{Off}$) at different bending diameter. (c) EPSC curves before and after bending.

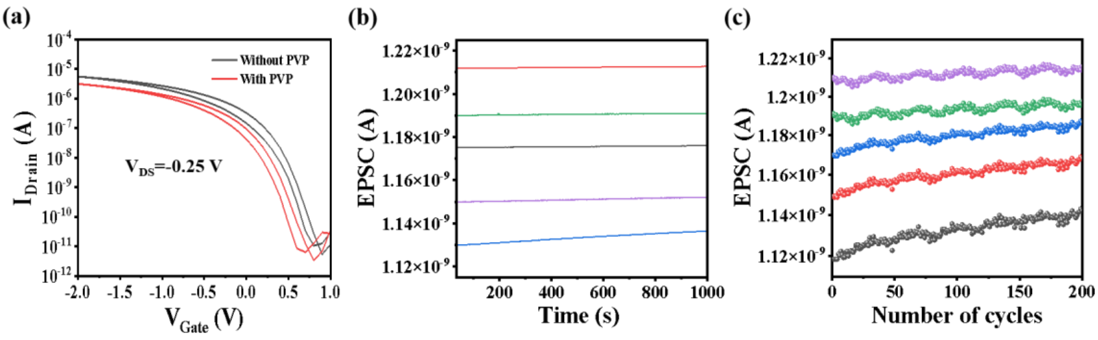


**Figure S14.** (a) Device transfer curves before and after spin-coating with PVP thin films. (b) The retention of the magnified 5 states measured up to 1000 s and (c) the cycling tests under illumination at various current states for the F8T2-SWCNT synaptic transistors without passivation by PVP thin films.

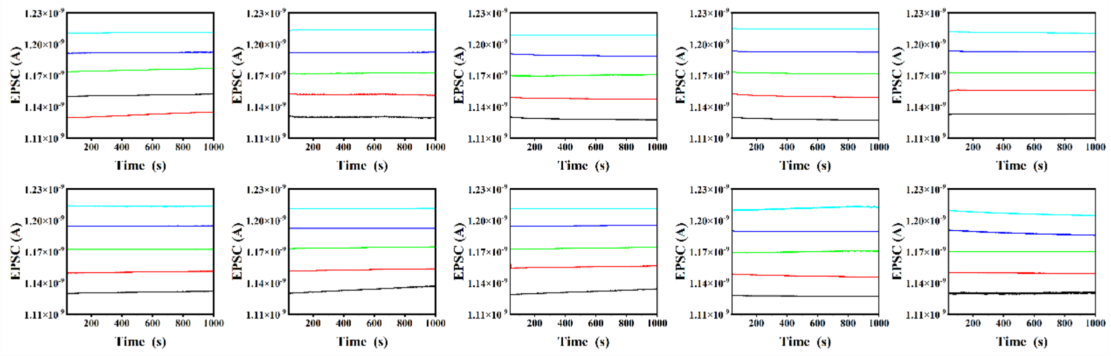


**Figure S15.** The retention properties of 10 devices randomly selected from the array.

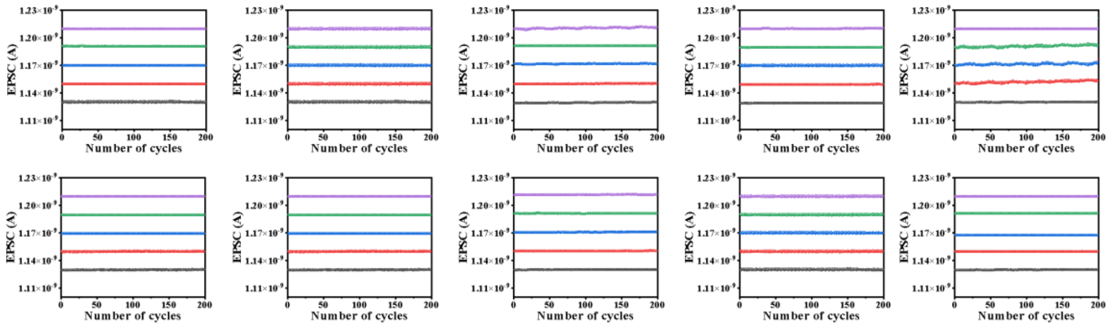


**Figure S16.** The cyclic stability of 10 devices randomly selected from the array.

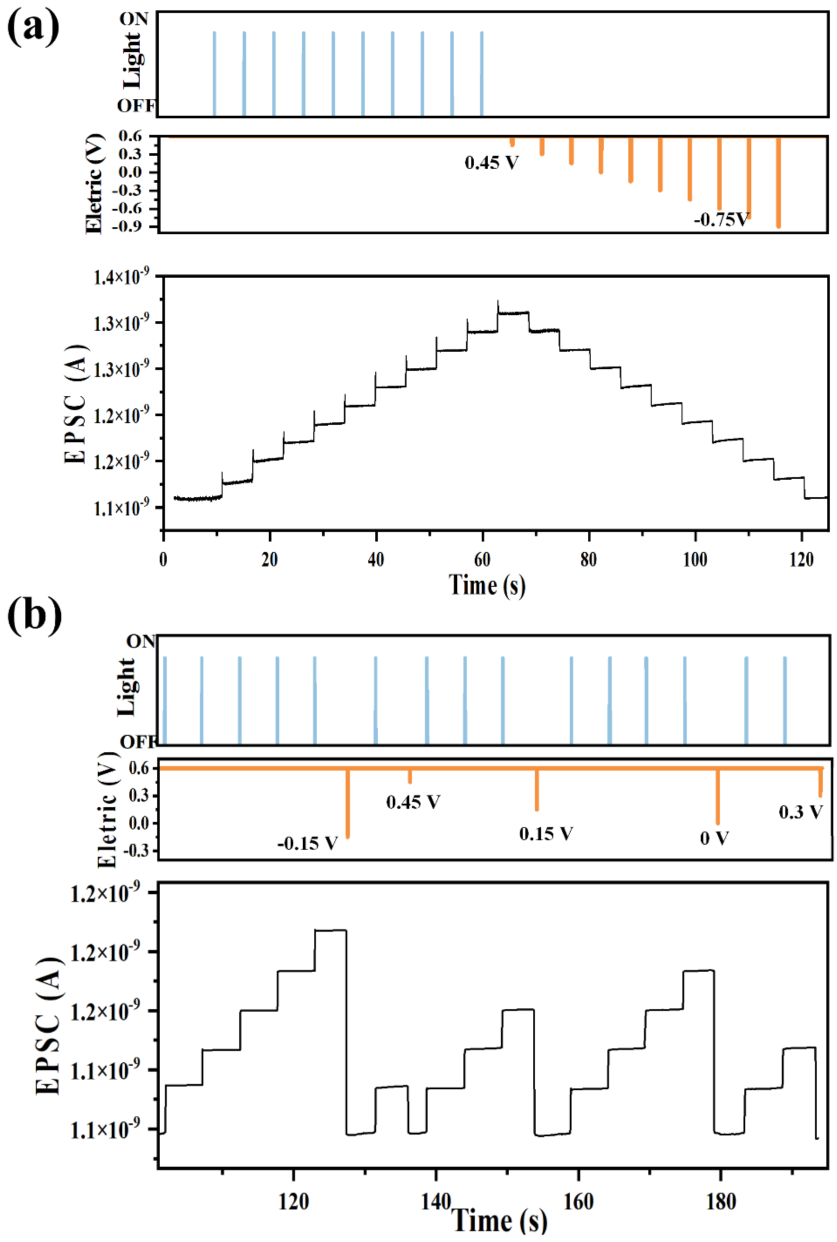


**Figure S17.** (a) Multiple optical programming and symmetric electrical erasure process. (b) Optical programming and electrical erasure processes for state 5-1-3-4-2. The optical and electrical pulse widths for the above experiment are 0.04 s and 0.1 s, respectively.

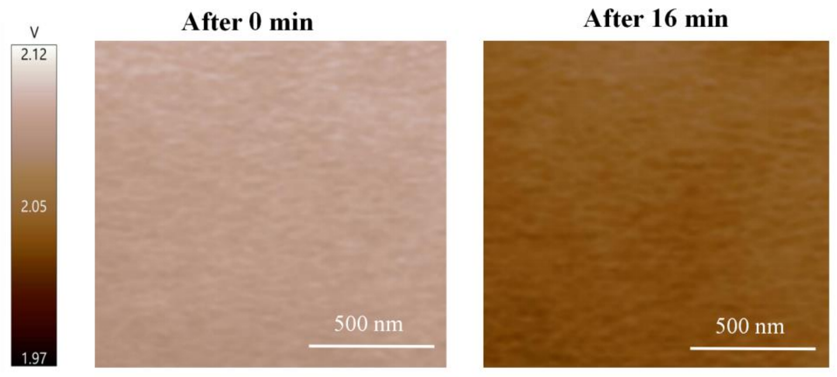


**Figure S18.** The surface potential of the SWCNT film after the light stimulation at 0 min and 16 min.

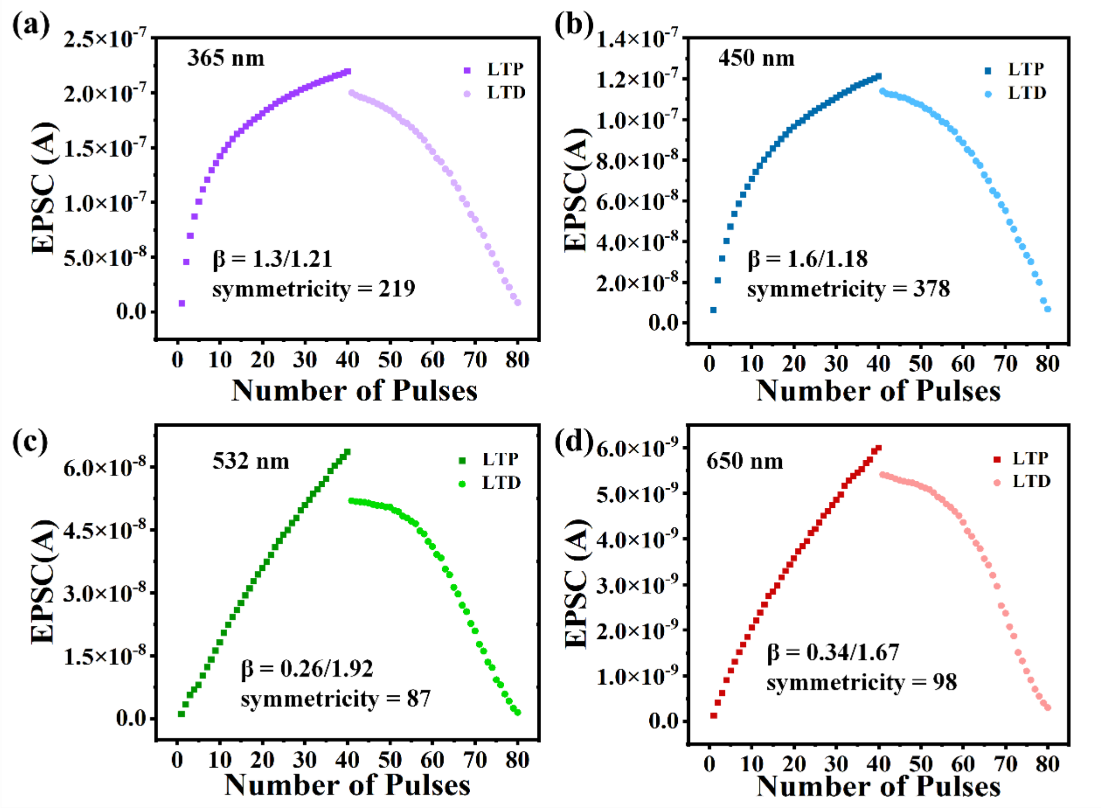


**Figure S19.** Change trend of LTP/LTD curves with wavelength with (a) 365 nm, (b) 450 nm, (c) 532 nm, (d) 650 nm.

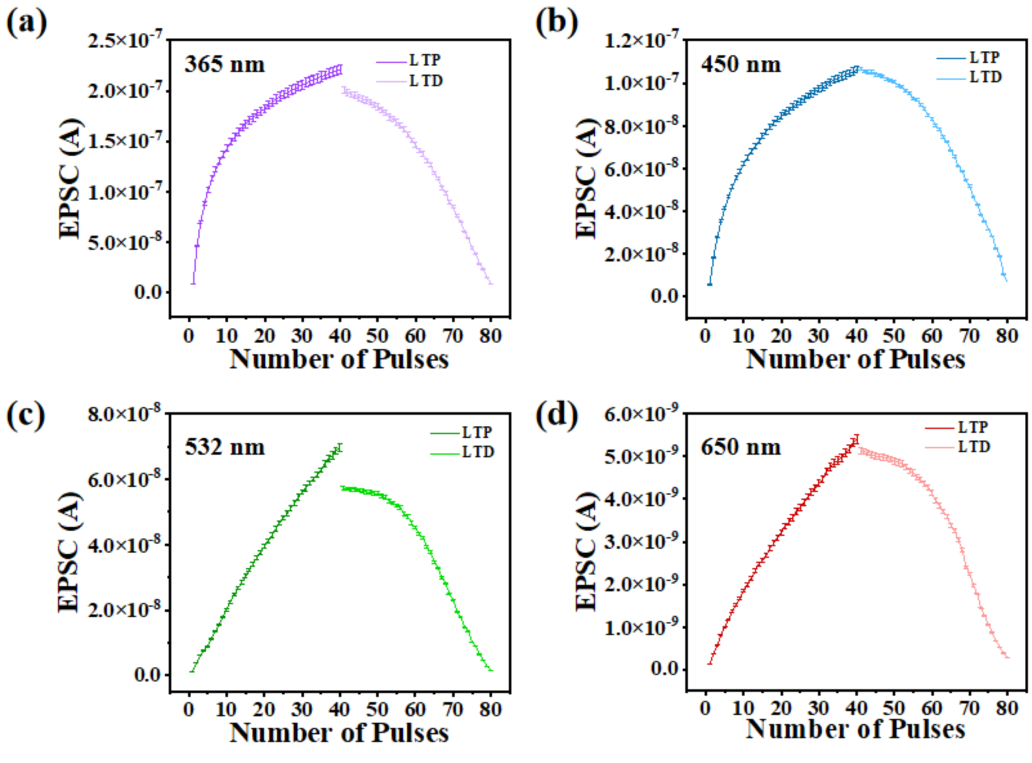


**Figure S20.** Cycle-to-cycle variations of LTP/D curves with wavelengths of (a) 365 nm, (b) 450 nm, (c) 532 nm and (d) 650 nm during 10 cycles. Average EPSC and standard deviation are represented.

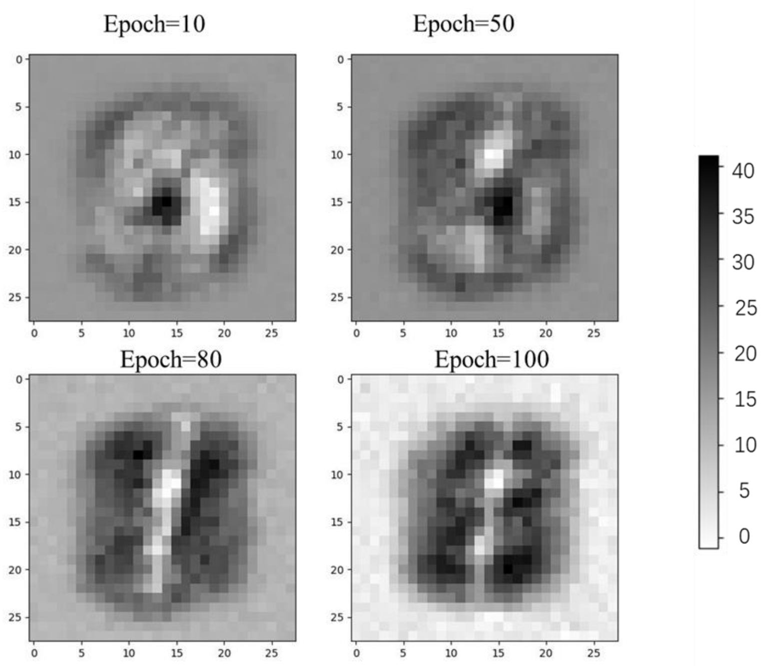


**Figure S21.** The mapping images of synaptic weights on the MNIST dataset.

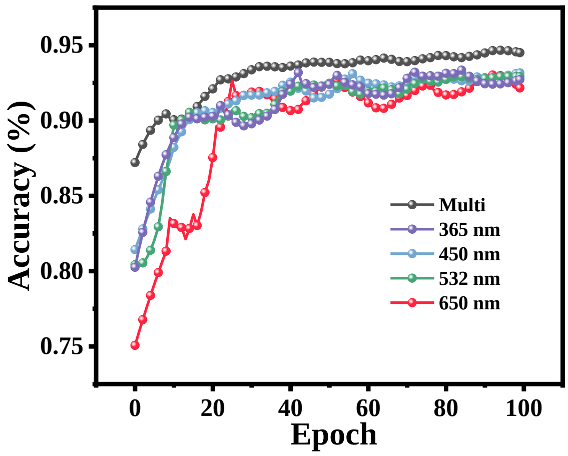


**Figure S22.** The recognition accuracy on the MNIST dataset.

**Table S1** Comparison of optoelectronic synaptic transistor device performance

| Substrate/ semiconductor | Operation Voltage (V) | On/off ratio | Light wavelength | Memory states | Power consumption | Ref |
|---|---|---|---|---|---|---|
| $Si/SiO_2/InGaCdO$ | ±20 | $10^8$ | 375 nm | NA | 0.14 nJ | [1] |
| $PET/MoS_2$ | ±8 | $10^4$ | 400 nm | NA | 4.1 pJ | [2] |
| $Si/SiO_2$/Pentacene | ±20 | $10^5$ | 365-850 nm | NA | 0.55 fJ | [3] |
| Si/OSC | ±20 | $10^5$ | 365 nm | NA | 0.11 fJ | [4] |
| $Si/SiO_2/MoS_2$ | ±9 | $10^6$ | NA | 6 | NA | [5] |
| $Cu/MoS_2$ | ±20 | $10^5$ | NA | 14 | NA | [6] |
| $Si/SiO_2/ReS_2$ | ±60 | $10^5$ | 405-785 nm | 128 | NA | [7] |
| $Si/SiO_2/WSe_2$ | ±10 | $10^6$ | 405 nm | 130 | NA | [8] |
| PI/SWCNT | ±1.5 | $10^6$ | 365-940 nm | 200 | 0.059 fJ | This work |

**Table S2** The initial setup of the algorithm simulation

| Dataset | Layer Size | | | Initial Weights | | Model Initialization | | Model Parameters | | | |
|---|---|---|---|---|---|---|---|---|---|---|---|
| | Input | Hidden | Output | Hidden | Output | Optimizer | Learning Rate | τ | $V_{th}$ | $T_{max}$ | $V_{DS}$ |
| Caltech face/motorbike | 160×250 | 400 | 2 | [0,1] | [0,50] | Adam | $10^{-6}$ | 1 | 100 | 20 | 0.25 |
| MNIST | 28×28 | 400 | 10 | [1,10] | [20,50] | SGD | $5 \times 10^{-3}$ | 1 | 100 | 20 | 0.25 |

## References


[1] H. Duan, K. Javaid, L. Liang, L. Huang, J. Yu, H. Zhang, J. Gao, F. Zhuge, T.-C. Chang, H. Cao, *physica status solidi (RRL)–Rapid Research Letters* **2020**, 14, 1900630.

[2] Y. Wang, J. Yang, W. Ye, D. She, J. Chen, Z. Lv, V. A. Roy, H. Li, K. Zhou, Q. Yang, *Advanced Electronic Materials* **2020**, 6, 1900765.

[3] J. Zhang, P. Guo, Z. Guo, L. Li, T. Sun, D. Liu, L. Tian, G. Zu, L. Xiong, J. Zhang, *Advanced Functional Materials* **2023**, 2302885.

[4] Q. Shi, D. Liu, D. Hao, J. Zhang, L. Tian, L. Xiong, J. Huang, *Nano Energy* **2021**, 87, 106197.

[5] Z. C. Zhang, Y. Li, J. Li, X. D. Chen, B. W. Yao, M. X. Yu, T. B. Lu, J. Zhang, *Advanced Functional Materials* **2021**, 31, 2102571.

[6] M. Jia, J. Yu, Y. Liu, P. Guo, Y. Lei, W. Wang, A. Yu, Y. Zhu, Q. Sun, J. Zhai, *Nano Energy* **2021**, 83, 105785.

[7] Q. Sun, M. Yuan, R. Wu, Y. Miao, Y. Yuan, Y. Jing, Y. Qu, X. Liu, J. Sun, *Advanced Materials* **2023**, 2302318.

[8] D. Xiang, T. Liu, J. Xu, J. Y. Tan, Z. Hu, B. Lei, Y. Zheng, J. Wu, A. C. Neto, L. Liu, *Nature communications* **2018**, 9, 2966.
